# Yerba Mate (*Ilex paraguariensis*) Ameliorates Experimental Autoimmune Encephalomyelitis by Modulating Regulatory T Cell Function

**DOI:** 10.3390/nu17050897

**Published:** 2025-03-04

**Authors:** Andrés A. Herrada, Francisca Rodríguez-Arriaza, Alexandra Olate-Briones, Sofía Albornoz-Muñoz, Jorge Y. Faúndez-Acuña, Victor Rojas-Henríquez, Ledaliz Retamal-Quinteros, Carolina Prado, Noelia Escobedo

**Affiliations:** 1 Lymphatic Vasculature and Inflammation Research Laboratory, Instituto de Ciencias Biomédicas, Facultad de Ciencias de la Salud, Universidad Autónoma de Chile, Talca 3467987, Chile; franciscarodriguezarriaza@gmail.com (F.R.-A.); alexandra.olate@uautonoma.cl (A.O.-B.); sofia.albornoz@alu.ucm.cl (S.A.-M.); jorge.faundez@alu.ucm.cl (J.Y.F.-A.); victor.rojas01@uautonoma.cl (V.R.-H.); ledaliz.retamal@gmail.com (L.R.-Q.); 2 Laboratorio de Neuroinmunología, Centro Científico y Tecnológico de Excelencia Ciencia & Vida, Fundación Ciencia & Vida, Avenida del Valle Norte #725, Huechuraba, Santiago 8580702, Chile; carolina.prado@uss.cl; 3 Facultad de Medicina y Ciencia, Universidad San Sebastián, Providencia, Santiago 7510156, Chile

**Keywords:** yerba mate, experimental autoimmune encephalomyelitis, regulatory T cells, inflammation

## Abstract

**Background/Objectives:** In Latin America, yerba mate (YM) is a popular infusion processed from the leaves and stems of *Ilex paraguariensis*. YM has been shown to have anti-inflammatory properties in several studies, although the effect of YM on multiple sclerosis (MS) remains elusive. The purpose of this study was to examine the effect of YM on the development of MS, by using the experimental autoimmune encephalomyelitis (EAE) mouse model while also evaluating its effect over infiltration of immune cells into the central nervous system (CNS) and regulatory T cell (Treg) function. **Methods**: YM or vehicle were administrated to mice daily by oral gavage for seven days prior to EAE induction and during the entire course of the disease. EAE score was recorded daily, and immune cell infiltration into the CNS was measured by flow cytometry and immunofluorescence. **Results**: Our results showed that YM administration decreases EAE symptoms and immune cell infiltration into the CNS, along with reducing demyelination, compared to the vehicle treatment. Moreover, an increase in the Treg population, immune cells capable of generating tolerance and decreased inflammation, was observed in mice receiving YM, together with improved Treg suppressive capabilities after YM treatment in vitro. **Conclusions**: In summary, we showed that YM promotes an immunosuppressive environment by modulating Treg function, reducing EAE symptoms and immune cell infiltration into the brain, and suggesting that YM consumption could be a good cost-effective treatment for MS.

## 1. Introduction

Multiple sclerosis (MS) is a chronic, autoimmune, demyelinating, and neurodegenerative disease of the central nervous system (CNS). MS is associated with a complex interplay between inflammatory and neurodegenerative processes, mostly attributed to pathogenic T and B cells. A key pathological feature of MS is the accumulation of demyelinating lesions or plaques, involving both resident cells as well as infiltrating immune cells [1]. Although the implication of infiltrating T cells and macrophages, which modulate the activity of microglia and neurons leading to demyelination and neuronal death have been well documented, the participation of other immune cell types either contributing or preventing neuroinflammation in MS has begun to be explored [2,3]. In this sense, several studies have shown alteration in regulatory T cell (Treg) function in MS patients [4,5]. Tregs are characterized by expressing the transcription factor FoxP3 and, after activation, they induce immunosuppression by different mechanisms, such as the expression of inhibitory molecules, the production of anti-inflammatory cytokines or immune suppressive molecules, and the suppression of a variety of immune cells, including B cells, T cells, natural killer cells, and antigen-presenting cells [6,7,8]. In fact, it has been demonstrated in the experimental autoimmune encephalomyelitis (EAE) model, a murine model of MS, that the transfer of Tregs ameliorated EAE symptoms, while Treg depletion exacerbates clinical manifestations, suggesting that modulating Treg function could be a good strategy to treat this autoimmune disorder [9,10,11,12].

*Ilex paraguariensis*, better known as yerba mate (YM) is a subtropical tree with multiple benefits due to its bioactive compounds. A hot or cold infusion of YM leaves and stems is traditionally consumed by South American countries, including Brazil, Argentina, Chile, Uruguay, and Paraguay [13]. Their beneficial properties include antioxidant activity, lowering cholesterol, preventing peroxidation, and reducing blood and tissue lipids, so its consumption is recommended in clinical conditions, such as obesity, hypertension, and diabetes [14]. YM has also immunomodulatory properties, with studies showing a reduction in proinflammatory cytokines produced by Th1/Th17 cells and decreased leukocyte migration and overall inflammation when YM is administrated in animal models of pleurisy and chronic smoke exposition [15,16]. Moreover, we have previously shown that YM reduces colitis severity and improves animal survival after dextran sodium sulfate (DSS)-induced colitis by promoting an immunosuppressive environment [17]. These potential therapeutic effects of YM have been attributed to saponins and polyphenols (chlorogenic acid and tannins) found in its leaves [18], flavonoids (quercetin, kaempferol, and rutin), xanthines (caffeine and theobromine), purine alkaloids (caffeic acid, 3,4-dicaffeoylquinic acid, and 3,5-dicaffeoylquinic acid), minerals (phosphorous, iron, and calcium), amino acids, and vitamins (C, B1, and B2) [18,19,20]. Even though several of these compounds have been associated with anti-inflammatory and immunosuppressive capabilities, whether YM consumption could reduce symptoms in pathological conditions, such as MS, is currently unknown.

The search for new therapeutic strategies to treat MS patients is crucial, not only because there is no cure for this pathology, but also because current treatments are mainly based on monoclonal antibodies that are expensive and difficult to access for low-income countries. Thus, the use of plant-based products that are inexpensive and with reduced or non-secondary effects emerges as a cost-effective alternative to treating MS. The aim of this study was to assess whether YM treatment provides protection against the development of EAE. Immune cell infiltration into the CNS and demyelination in animals receiving YM or a vehicle were evaluated. Considering that YM consumption changes the composition of the mouse microbiota [17,21], fecal microbiota transfer experiments were performed to analyze the importance of gut microbiota changes triggered by YM consumption over EAE symptoms. Finally, since Tregs are important immunosuppressive cells capable of decreasing inflammation, the effect of YM on this immune cell population was analyzed in vivo and in vitro.

## 2. Materials and Methods

### 2.1. Mice

Male and female C57BL/6/J wild-type (*WT*) and congenic CD45.1^+^ FoxP3^GFP^ mice were obtained from Jackson Laboratories. Eight-to-twelve-week-old male and female mice were used in this study. Animals were housed in temperature- and humidity-controlled rooms, maintained on a 12 h light/12 h dark cycle (lights on at 7:00 h), received water and food ad libitum, and were provided with environmental enrichment (Eco-BeddingZuPET provided by Animal Care, Santiago, Chile). All animal experiments and procedures were carried out in accordance with approved Institutional Animal Care and Use Committee at the Universidad Autónoma de Chile (protocol codes BE04-20 and BE01-23). Animals were maintained according to the “Guide to Care and Use of Experimental Animals, Canadian Council on Animal Care”.

### 2.2. Yerba Mate Preparation

The YM solution was made daily by dissolving dry extract of *Ilex paraguariensis* leaves (Pajaritos, Paraguay) in hot water at 60 °C, utilizing a homogenizer. Previously, our group analyzed a sample of this lot, and the results were published [17]. The YM extract contained theobromine (892 ± 55 mg/Kg), rutin (486 ± 16 mg/Kg), chlorogenic acid (2461 ± 122 mg/Kg), quinic acid (18 ± 4 mg/Kg), quercetin (39 ± 7 mg/Kg), and caffeine (2388 ± 189 mg/Kg). The solution was filtered using a 0.2 µm filter (Thermo Scientific, Waltham, MA, USA) before being added to the cell culture. For in vitro studies, and based on our previous study, 50 µg/mL of YM or vehicle was used [17]. For in vivo experiments, 1 g of the leaf dry extract (Pajaritos, Paraguay) was dissolved in 8 mL of hot water (60 °C) and allowed to cool, then filtered and administered by oral gavage (0.025 g per mouse) daily for seven days before EAE induction and during the entire procedure. At this dose, animals do not have any sign of cytotoxicity, and this is consistent with studies showing that animals receiving a higher dose of YM (0.06 g per mouse) for over 60 days showed no toxicological effects [22]. Control mice received only vehicle (water heated to 60 °C, allowed to cool, and then filtered). In all instances, the YM solution (and vehicle) was made fresh daily to preserve its properties.

### 2.3. EAE Induction and Clinical Assessment

Eight-to-twelve-week -old C57BL/6 mice were injected s.c. in the flank with 50 mg MOG_35–55_ peptide emulsified in complete Freund’s adjuvant (CFA) (Sigma-Aldrich, St. Louis, MO, USA), supplemented with heat-inactivated *Mycobacterium tuberculosis* H37 Ra (Becton-Dickinson, Franklin Lakes, NJ, USA). At the time of sensitization and 48 h later, mice received i.p. injection of 500 ng of Pertussis toxin (Thermo Fisher). Following sensitization, clinical manifestations of the disease were evaluated daily based on the subsequent scoring criteria: 0, no detectable signs of EAE; 1, flaccid tail; 2, hind limb weakness or abnormal gait; 3, complete hind limb paralysis; 4, paralysis of fore and hind limbs; and 5, moribund or dead. To prevent unnecessary animal suffering, mice severely affected by the disease were euthanized with the supervision of a veterinarian. At day 15 after EAE induction, a group of animals were anesthetized and sacrificed by transcardial perfusion with phosphate buffered saline 1X (PBS) and the tissues were harvested and analyzed, as described below.

### 2.4. Immunohistochemistry

Brains were washed with cold PBS and fixed in 4% paraformaldehyde (Sigma-Aldrich) in PBS at 4 °C for 48 h. Samples were then washed in PBS and dehydrated on a sucrose gradient with 15% sucrose in PBS, followed by 30% sucrose overnight at 4 °C. The next day, the tissues were embedded in Tissue-Tek OCT and stored at −80 °C before sectioning. Brain samples were sectioned at 100 µm with a cryostat and immunostained as free-floating cryosections. Briefly, samples were slightly permeabilized in PBS and 0.5% Triton X-100 for 15 min, blocked with a mix of 5% donkey serum (Jackson Immuno Research, West Grove, PA, USA), 1% bovine serum albumin, 0.05% sodium azide, and 0.1% Triton X-100 in PBS for 3 h, and incubated overnight at room temperature with anti-CD3 (clone 17A2, BioLegend, San Diego, CA, USA) at 1:200 dilution or anti-B220 (clone RA3-6B2, BioLegend) at 1:200 dilution. The next day, samples were washed with PBS and 0.1% Triton X-100 five times for 15 min, and then the samples were incubated with the respective secondary antibodies conjugated with fluorophores at 1:250 dilution for 3 h in darkness. Samples were washed with PBS and 0.1% Triton X-100 five times for 15 min, mounted on a coverslip with VECTASHIELD antifade mounting medium with DAPI (Vector Laboratories, Burlingame, CA, USA) and sealed with nail polish. Images were obtained using a confocal microscope Leica Stellaris 5 (Leica Microsystems, Wetzlar, Germany). The numbers of T cells (CD3^+^) and B cells (B220^+^) were manually quantified in a blinded manner using ImageJ software (version 1.53t) and quantified from the photos obtained in the confocal microscope of brain coronal sections in the area close to the dorsal third ventricle and at a magnitude of 63X. After performing a z-stack and a maximum projection of the full stack, the numbers of cells were quantified on each photo (5 images per animal), averaged, and plotted as a single point per animal, with three animals per group.

### 2.5. Histological Analysis of Spinal Cords

For histopathological analysis, the demyelinated area in spinal cord was measured using Luxol Fast Blue staining. Once the animals were perfused, the entire spinal column, including the vertebrae and enclosed spinal cord, were removed, and the epaxial muscles were manually dissected off and fixed in PFA4% for 48 h at 4 °C. Then, the spines were briefly washed with PBS and placed in 14% EDTA (Sigma ED-EDTA, pH 7.3) for decalcification with continuous shaking at 4 °C for 2 weeks and with changes to fresh 14% EDTA solution every 3 to 4 days. Then, the entire spinal column was manually cleaned and cut into 3 transverse segments along the anterior–posterior axis, maintaining the appropriate orientation. Tissues were then sequentially dehydrated using alcoholic solutions (ethanol 70%, ethanol 90%, and ethanol 100%) and Neo-Clear™ Xylene Substitute (Sigma-Aldrich); embedded in paraffin (Paraplast-Plus, Leica Biosystems, Wetzlar, Germany), and samples were sectioned at 10 μm thickness using a microtome (Biobase Biozone Co., Jinan, China). Then, the sections were dewaxed using Neo-Clear (Sigma, St. Louis, MO, USA) and hydrated and stained using Luxol Fast Blue Stain Kit (Abcam, Cambridge, UK) to evaluate demyelination, according to the manufacturer’s instructions. Images were acquired using an Olympus BX51 microscope (Olympus America, Inc., Center Valley, PA, USA). Demyelination was assessed in a blinded manner by detecting white matter areas that lacked the Fast Blue staining by using the FIJI software (Image J, version 2.14.0/1.54f) and following the protocol from Ucciferri et al. with modifications [23]. Briefly, the dorsal and anterolateral sections of the spinal cord were delineated to represent the total white matter area. The percentage of myelination was calculated by dividing the combined area of the Fast Blue-stained foci in both sections by the total white matter area. The demyelination percentage was then determined by subtracting the myelination percentage from the total white matter area. The area of demyelination was measured in at least 3 photos per animal, and the average area of each animal was plotted, with 3 animals per group.

### 2.6. In Vitro Treg Studies

For in vitro Treg differentiation assays, splenic CD4^+^ T cells from FoxP3^GFP^ animals were first pre-enriched by negative selection by using MACS (Miltenyi Biotec, Bergisch Gladbach, Germany), and then naïve (CD4^+^CD62L^+^CD44^−^CD25^−^ FoxP3-GFP^−^) T cell isolation was achieved by cell sorting using a FACS Aria II (BD, Franklin Lakes, NJ, USA), obtaining purities over 98%. Naïve T cells were cultured at 37 °C with 5% CO_2_ in complete RPMI medium (10% FBS, 1% HEPES (Gibco, Frederick, MD, USA) and 1% penicillin/streptomycin solution (ThermoFisher, USA)), with plate-bound anti-CD3 (50 ng/well), soluble anti-CD28 (2 µg/mL), 5 ng/mL TGF-β1, and 10 ng/mL IL-2, with YM (50 µg/mL) or vehicle for 4 days. Treg differentiation was evaluated by the expression of FoxP3-GFP in CD4^+^ T cells by flow cytometry. For in vitro suppression assays, spleen from congenic CD45.1^+^ FoxP3^GFP^ mice were obtained and Tregs (CD4^+^FoxP3-GFP^+^) were sorted using a FACS Aria II (BD, Franklin Lakes, NJ, USA). Tregs were activated in complete RPMI medium with 50 ng of plate-bound anti-CD3 and 2 μg/mL soluble anti-CD28 in the presence of YM (50 µg/mL) or vehicle at 37 °C with 5% CO_2_. After 2 days, activated Tregs were washed and co-cultivated at a ratio of 1:1 with Cell Trace Violet (CTV)-stained naïve CD45.2^+^CD4^+^ T-cells sorted from *WT* mice in the presence of anti-CD3 and anti-CD28 antibodies. After 72 h, the extent of naïve T-cell proliferation was determined as the dilution of CTV-associated fluorescence in the CD45.1^−^CD45.2^+^CD4^+^ population by flow cytometry.

### 2.7. Flow Cytometry

Mice were euthanized, and then peripheral lymph nodes (axillary and inguinal) and the spleen were manually isolated and processed for flow cytometry. Spleens and lymph nodes were minced until they reached a cell suspension before being filtered, and then, for spleen, red blood cells were lysed using ACK buffer (ammonium chloride 0.15 M; potassium bicarbonate 0.01 M; disodium EDTA 0.1 mM; pH 7.2–7.4). Cells were counted and stained in PBS containing 2% (*w*/*v*) BSA. 7AAD (Cat No 420403, Bio-Legend) was used for dead-cell exclusion. The following fluorescent conjugate-labelled antibodies were used: anti-CD45 (clone 30-F11), anti-CD45.1 (clone A20), anti-CD45.2 (clone 104), anti-CD4 (clone GK-1.5), anti-CD8 (clone 2.43), anti-CD19 (clone 1D3), anti-CD3 (clone 17A2), anti-PD-1 (clone 29.F.1A12), and anti-CTLA-4 (clone UC10-4139). These antibodies were purchased from Biolegend. Anti-CD25 (clone PC61.5) was purchased from Cytek (Fremont, CA, USA) and anti-FoxP3 (clone FJK-16) and anti-TCRβ (clone H57-597) were purchased from eBioscience (San Diego, CA, USA). For FoxP3 immunostaining, cells were first labelled with antibodies specific to cell-surface markers, then fixed and permeabilized with a FoxP3 Fixation/Permeabilization kit (eBioscience) and stained with the anti-FoxP3 antibody. Samples were analyzed by flow cytometry using a BD FACSCanto II instrument (BD Bioscience, Franklin Lakes, NJ, USA) and data were analyzed using FlowJo version X.0.7 (Tree Star, Inc., San Carlos, CA, USA).

### 2.8. Fecal Microbiota Transplantation (FMT)

FMT was performed as described previously [24]. Briefly, feces were aseptically collected from mice treated with YM or vehicle at day 14, then weighed, macerated, and dissolved in glycerol buffer (10%) in PBS at concentration of 1g of feces/10 mL of glycerol buffer. Eight-to twelve-week-old healthy *WT* recipient animals were treated with a mixture of broad-spectrum antibiotics containing ampicillin (1 mg/mL), metronidazole (1 mg/mL), neomycin (1 mg/mL), and vancomycin (0.5 mg/mL) in water for 7 days as described, and microbiota depletion was checked daily in the feces by qPCR using the conserved 16S rRNA-specific primer pair [25]. After two days of resting with regular water (wash out), recipient *WT* mice received intragastric administration of 200 µL of fecal suspension to each mouse, with care taken to avoid regurgitation. FMT was repeated twice every other day and two days after the last FMT, EAE was induced as described above.

### 2.9. Statistical Analysis

Statistical analysis was performed using Prism (GraphPad Software, version 8.0.2, San Diego, CA, USA). The data are expressed as the means ± standard error of the mean (SEM). Data distributions were tested for normality using the Shapiro–Wilk normality test (https://www.statskingdom.com/320ShapiroWilk.html, (accessed on 13 September 2024)). The significant differences between different data were calculated using an unpaired two-tailed *t*-test (for two groups) or one-way ANOVA (for more than two groups), followed by Tukey’s multiple comparison test. For grouped analysis, two-way ANOVA followed by a Sidak multiple comparison was used. EAE-free curves were calculated using a log-rank (Mantel–Cox) test. The overall *p* value < 0.05 was considered statistically significant; * *p*  ≤  0.05, ** *p*  ≤  0.01, *** *p*  ≤  0.001 and **** *p*  ≤  0.0001.

## 3. Results

### 3.1. Yerba Mate Consumption Reduces EAE Symptoms

**Figure 1:**
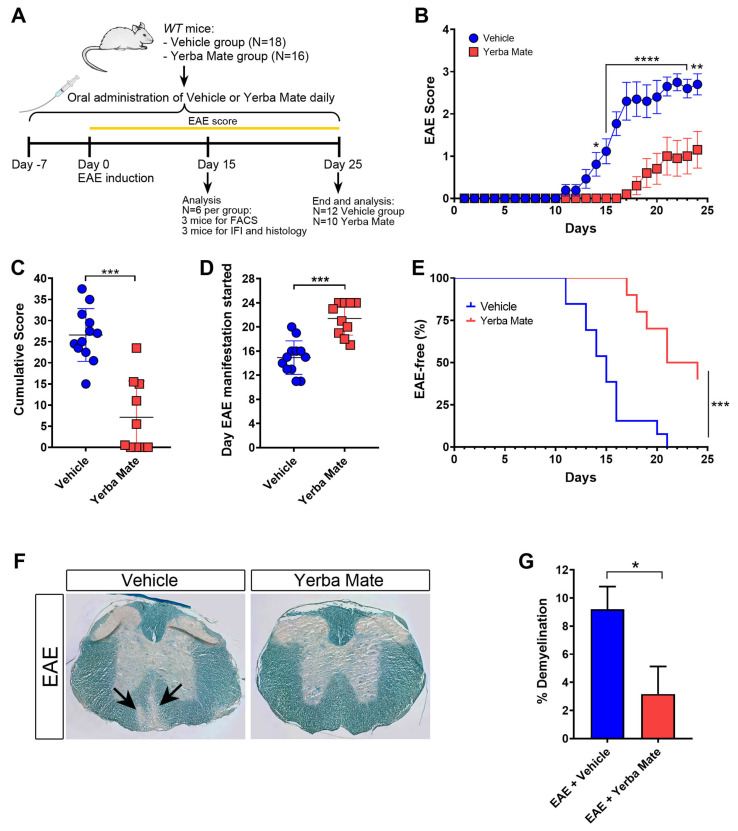
Yerba mate decreases EAE symptoms and neuronal damage. (**A**) Schematic illustration of the experimental design. *WT* mice received yerba mate (0.025 g per mouse) or vehicle by oral gavage seven days before EAE induction and until the end of the experiment. Animals were immunized subcutaneously with MOG 35-55-peptide emulsified in CFA and i.p injected with Pertussis toxin at day 0 and day 2 after EAE induction. Score was monitored daily for 25 days, and at day 15 a group of animals were sacrificed and immune cell infiltration and demyelination were evaluated by immunofluorescence, flow cytometry, and Luxol Fast Blue staining. (**B**) EAE clinical score; (**C**) cumulative score; (**D**) day that clinical symptoms started; (**E**) Kaplan–Meier curve graphing the percentage of EAE-free mice over time. Blue circles: vehicle; red squares: yerba mate. Data are plotted as mean ± SEM from 12 (vehicle group) and 10 (yerba mate group) mice from three independent experiments. * *p* < 0.05; ** *p* < 0.01; **** *p* < 0.0001 by two-way ANOVA followed by Sidak’s multiple-comparison test (**B**); *** *p* < 0.001 by unpaired *t*-test (**C**,**D**), and *** *p* < 0.001 by log-rank (Mantel–Cox) test (**E**). (**F**,**G**) Demyelination pathological analysis of spinal cord from animals treated with vehicle or yerba mate on day 15 after EAE induction. Representative images of transverse sections of spinal cords stained with Luxol Fast Blue showing areas of demyelination in the EAE group treated with vehicle (arrows) (**F**) and percentage of demyelination in the experimental groups (**G**) are shown. Data from *n* = 3 mice per group are shown. Bars represent mean ± SEM. * *p* < 0.05 by unpaired *t*-test (**G**).

YM consumption has been shown to reduce inflammation in different animal models [15,16,17], but the potential beneficial effect of YM administration on EAE has not been yet addressed. Thus, we decided to analyze the effect of YM consumption in the initiation and progression of EAE, the most common animal model of MS, characterized by brain and spinal cord inflammation and demyelination. A daily oral gavage solution of YM (0.025 g per mouse) or vehicle was administered 7 days before EAE induction and until the end of the experiment (Figure 1A). While animals treated with vehicle developed EAE symptoms in a similar way to that which we have previously reported [26], we observed a drastic reduction in EAE symptoms in mice receiving YM (Figure 1B). Additionally, a significant reduction in the cumulative score and a delay in the appearance of the first EAE symptoms were also evident (Figure 1C,D). Moreover, in contrast to the vehicle group, the YM group had a higher percentage of healthy animals (Figure 1E). Luxol Fast Blue staining was performed to visualize the myelin in histological sections of the spinal cord at day 15 after EAE induction, and the area of demyelination was measured. Histological analysis of spinal cords revealed a reduction in myelin loss in the YM-treated group compared to the vehicle group (Figure 1F,G). In fact, animals suffering EAE that received YM looked healthier compared to vehicle-treated EAE mice (Supplementary Video S1). All these results suggest that YM decreases EAE symptoms and incidence, together with diminished demyelination, which, overall, positively impacts animal behavior.

### 3.2. Decreased Immune Cell Infiltration in Mice Receiving YM

**Figure 2:**
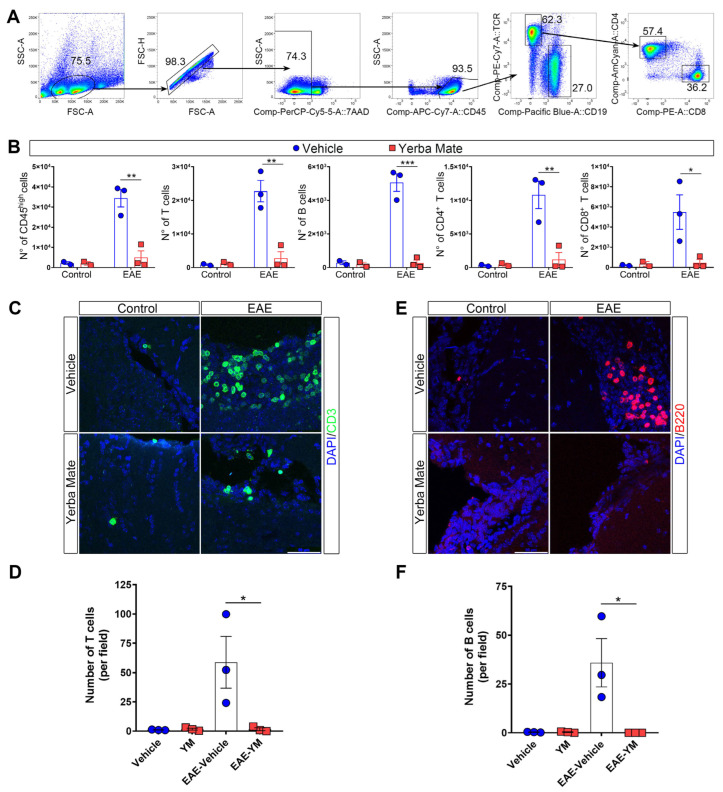
Yerba mate reduces immune cell infiltration into the CNS. (**A**) Flow cytometry gating strategy to evaluate myeloid cells (live, CD45^+^), total T cells (CD45^+^TCRβ^+^CD19^−^), B cells (CD45^+^TCRβ^−^CD19^+^), CD4 T cells (CD45^+^TCRβ^+^CD4^+^), and CD8 T cells (CD45^+^TCRβ^+^CD8^+^). (**B**) Graphs showing the number of the different immune cell populations analyzed by flow cytometry from the brains of control animals or animals at day 15 after EAE induction treated with vehicle or yerba mate. Blue circles: vehicle; red squares: yerba mate. Data from *n* = 3 mice per group are shown. Bars represent mean ± SEM (**C**–**F**). Representative brain cryosections (**C**,**E**) immunostained with specific antibodies for T cells (CD3, green) (**C**) or B cells (B220, red) (**E**), of animals from the vehicle (upper panels) or yerba mate (lower panels) groups either untreated (control, left panels) or at day 15 after EAE induction (EAE, right panels). Images were captured using a confocal microscope at 63× magnification, performing a z-stack every 0.5 μm and then a maximum projection of the full stack (**D**,**F**). Scale bar is 50 μm. CD3-positive cells (**D**) or B220-positive cells (**F**) were quantified based on an image’s z-stack and the maximum projection of each image. Each point represents one animal from the average of five images analyzed per animal. Bars represent mean ± SEM. * *p* < 0.05; ** *p* < 0.01; *** *p* < 0.001 by one-way ANOVA followed by Tukey’s post-test.

Since peripheral immune cell infiltration into the CNS is a key step in promoting demyelination and neuroinflammation during EAE, we decided to evaluate immune cell infiltration in the brains of animals receiving YM or vehicle once EAE symptoms appeared. Thus, mice receiving YM or vehicle were induced or not with EAE, and at day 15 after EAE induction, the animals were sacrificed and their brains were obtained and processed to analyze immune cell infiltration by flow cytometry and immunofluorescence. The gating strategy is illustrated in Figure 2A. We found that, while YM consumption does not impact immune cell populations in controls animals, there is a drastic reduction in the number of infiltrating immune cells in the brains of animals suffering EAE and receiving YM, compared to the EAE vehicle group (Figure 2B). Total T cells, CD4^+^ T cells, CD8^+^ T cells and B cells, were significantly reduced in the EAE YM group compared to the EAE vehicle group (Figure 2B). Next, to corroborate these results, we performed immunostaining of T and B cells in coronal sections of brains of controls animals and animals suffering EAE and receiving either YM or vehicle (Figure 2C–F). Reduced T cell infiltration was evident in the brains of EAE YM group compared to the EAE vehicle group, supporting a reduction in immune cell infiltration in YM-treated animals (Figure 2C,D). Additionally, B cell numbers were also reduced in the EAE YM group compared to the EAE vehicle group (Figure 2E,F). Together, these results suggest that YM treatment reduces immune cell infiltration into the CNS during EAE, decreasing demyelination in these animals.

### 3.3. Changes in Gut Microbiota Do Not Explain the Protective Capacity of YM in EAE

**Figure 3:**
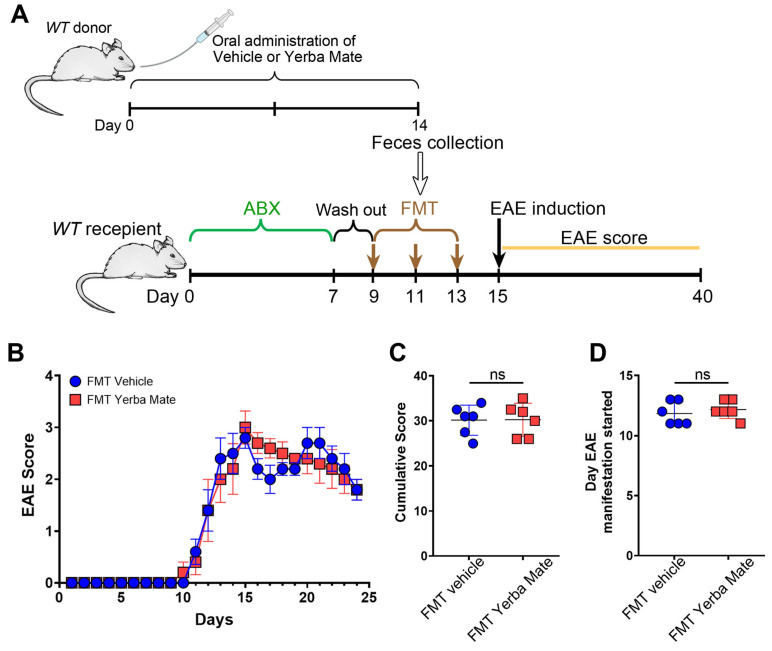
The protective effect of yerba mate against EAE does not depend on the gut microbiome. (**A**) Schematic illustration of the experimental design. Donor WT mice received yerba mate (0.025 g per mouse) or vehicle by oral gavage for 14 days. On day 14, feces were collected and FMT was performed in recipient mice that were previously treated with a cocktail of antibiotics for 7 days and then with regular water for another 2 days. FMT was repeated twice every other day and EAE was induced 2 days after the last transplant, and clinical score was monitored for 24 days. (**B**) Clinical EAE score, (**C**) cumulative score, and (**D**) the day that clinical symptoms started are shown. Blue circles: vehicle; red squares: yerba mate. Data are plotted as mean ± SEM from 6 mice per group from 2 independent experiments. ns: not significant.

The gut microbiota, which includes bacteria, archaea, fungi, and viruses, is responsible for regulating immune homeostasis and providing essential health benefits [27]. Alteration in gut microbiota, known as dysbiosis, are found in MS patients and animal models of this disease [28,29]. Since we and others have found that YM consumption modulates gut microbiota composition [17,30], we evaluated whether the protective effect of YM in EAE is explained by the changes induced in the gut microbiota. Thus, we performed FMT experiments, where recipient animals received feces from mice treated with YM or vehicle for 14 days, and then EAE was induced (Figure 3A). Interestingly, even while bacteria from the feces effectively repopulated the gut of recipient’s animals, we did not observe any protection regarding clinical symptoms, cumulative score, or disease onset in the animals’ receiving feces from YM-treated mice compared to animals receiving feces from vehicle control group (Figure 3B–D). Based on these results, it seems that changes in the gut microbiota do not explain the protective effect of YM over EAE development.

### 3.4. YM Consumption Increases the Treg Population in Secondary Lymphoid Organs (SLOs)

**Figure 4:**
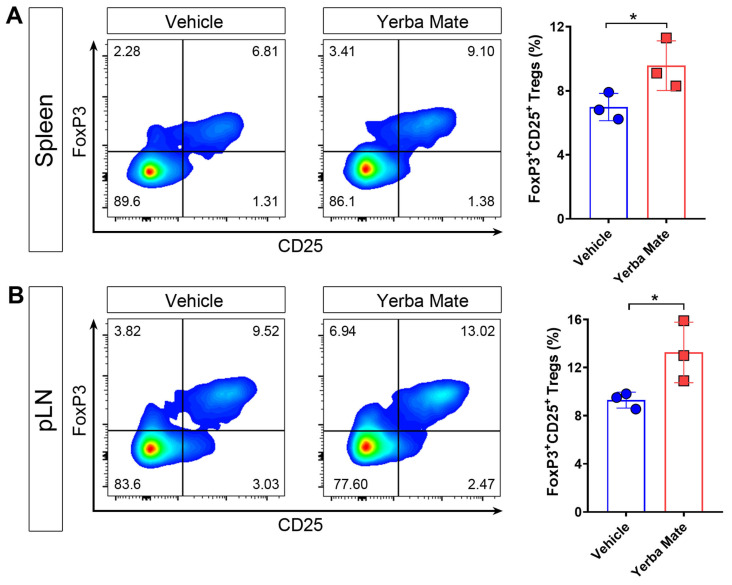
Yerba mate increases the peripheral Treg population in vivo. Animals receiving yerba mate or vehicle for 7 days were sacrificed and Treg frequency was analyzed in the peripheral lymph nodes and spleen. (**A**) Representative pseudo-color dot-plots of Tregs (CD4 ^+^CD3^+^CD25^+^ FoxP3^+^) in the spleen (left) and quantification (right) are shown. (**B**) Representative pseudo-color dot-plots of Tregs (CD4 ^+^CD3^+^CD25^+^ FoxP3^+^) in peripheral lymph nodes (left) and quantification (right) are shown. Data are plotted as mean ± SEM from 3 mice per group. * *p* < 0.05 by unpaired *t*-test.

It is well known that Tregs are key players in controlling autoimmune damage during EAE [10,12,31]. Due to our results about reduced symptoms and decreased peripheral immune cell infiltration into the CNS in EAE-induced mice and treated with YM, we considered whether YM affects Treg numbers in vivo. Thus, healthy animals received YM or vehicle for one week and then the Treg population was evaluated in SLOs by flow cytometry. Our results showed that after one week of YM treatment, Treg frequency increased in spleen and peripheral lymph nodes compared to the vehicle group, suggesting that YM modulates the Treg population in vivo (Figure 4A,B).

### 3.5. YM Directly Modulates Treg Suppressive Function In Vitro

**Figure 5:**
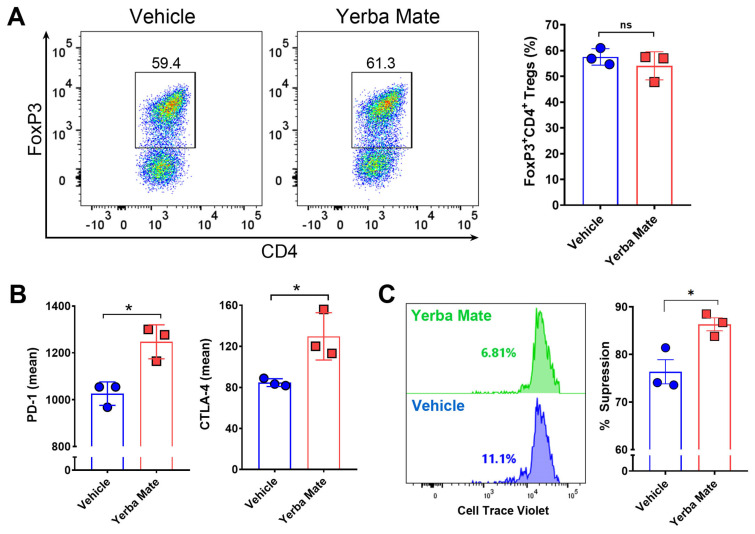
Yerba mate increases Treg suppressive function in vitro. Naïve T cells (CD4^+^CD62l^+^CD44^−^CD25^−^FoxP3-GFP^−^) were purified from the spleen of CD45.1^+^ FoxP3-GFP mice and stimulated with anti-CD3 (50 ng/well) and anti-CD28 (2 μg/mL) antibodies under Treg differentiation conditions in the presence of yerba mate (50 µg/mL) or vehicle and evaluated by flow cytometry 72 h later. (**A**) Representative density plots (left) and quantification of Treg frequency (right) are shown. (**B**) Levels of suppressive molecules expressed in the Treg population (CD4^+^CD25^+^FoxP3-GFP^+^). (**C**) In vitro suppression assays. Tregs (CD4^+^FoxP3-GFP^+^) purified from the spleen of CD45.1^+^ FoxP3-GFP mice were activated with plate-bound anti-CD3 (50 ng) and soluble anti-CD28 (2 μg/mL) with yerba mate (50 µg/mL) or vehicle. After 2 days, activated Tregs were washed and co-cultivated at a ratio of 1:1 with Cell Trace Violet (CTV)-stained naïve CD4^+^ T cells sorted from CD45.2^+^ WT mice in the presence of anti-CD3 and anti-CD28 antibodies. After 72 h, naïve T-cell proliferation was evaluated by dilution of CTV-associated fluorescence in the CD45.1-CD45.2+CD4+ population. Representative histograms of CTV dilution profiles are shown (left). Numbers on the histograms represent the percentage of T effector cells displaying CTV dilution. In the right panels, the extent of suppression is quantified as the percentage of inhibition of T effector proliferation relative to maximal proliferation (No Treg), where the percentage of suppression is 0. Data are plotted as mean ± SEM from three independent experiments. ns: not significant; *: *p* < 0.05; by unpaired *t*-test.

Because of our results showing an increased population of Tregs in SLOs from animals receiving YM, we hypothesized that YM can directly modulate Treg differentiation and/or function. Thus, we evaluated the effect of YM over Treg differentiation and function in vitro. First, we analyzed the effect of YM over Treg differentiation. Naïve T cells were purified from FoxP3-GFP mice and cultured under Treg differentiation conditions with YM (50 µg/mL) or vehicle for 72 h, and then FoxP3 expression was analyzed by flow cytometry. Interestingly, we did not observe any effect of YM over Treg differentiation in vitro (Figure 5A). However, under this condition, the presence of YM increased the expression of PD-1 and CTLA-4, two important proteins involved in the suppressive activity of Tregs (Figure 5B). Thus, we next evaluated the effect of YM over the suppressive activity of Treg in vitro, by culturing purified Tregs with YM or vehicle, washing the cells, and then co-culturing them with CTV-stained naïve CD4^+^ T cells. Our results showed that YM increased the Treg suppressive activity when compared to the vehicle control (Figure 5C). These results suggest that YM is capable of modulating Treg function in vitro, increasing the expression of PD-1 and CTLA-4 and improving their suppressive activity. Overall, our results suggest that YM can reduce EAE symptoms and decrease immune cell infiltration into the CNS, and this therapeutic effect is not explained by changes in the gut microbiota composition, but most likely functions by directly modulating Treg suppressive activity.

## 4. Discussion

MS is an autoimmune demyelinating disease of the CNS characterized by neurodegenerative and inflammatory processes, primarily caused by pathogenic T and B cells. In addition to the deleterious effect of autoreactive T and B cells, the Treg population is also disturbed in MS patients, with studies showing reduced numbers of this population in peripheral blood from MS patients, together with decreased suppressive capabilities compared to healthy controls [5,32,33,34]. Accordingly, novel Treg therapies for the treatment of MS are under investigation [35,36]. Thus, the development of new strategies to boost Tregs number and function could be a good therapeutic approach to reduce MS symptoms.

YM has been recognized for its biological benefits, which could be explained in part by the modulation of specific immune cell types [17,37]. YM’s effect on T cell activation has been reported, although no further studies regarding modulation of specific T cell subtypes by YM have been investigated [37]. Here, we observed that YM consumption reduces EAE symptoms and immune cell infiltration into the CNS, together with increasing the Treg population in vivo and enhancing its suppressive activity directly in vitro. Even though YM modulates gut microbiota composition [17,38], we did not observe any protective effect in gut microbiota from YM-treated mice in our FMT experiments, suggesting that YM acts directly over Treg. Interestingly, by using the same batch of YM that was used in this study, we found that chlorogenic acid, a significant biologically active dietary polyphenol, was the predominant bioactive compound present in our extract (2461 ± 122 mg/Kg) [17]. This compound has been described to have several therapeutic functions, such as mitigating inflammation caused by type 1 diabetes in a rat model of this disease and decreasing allergic rhinitis-related symptoms in mice [39,40]. Additionally, it has been recently described that chlorogenic acid increases the Treg population in racehorses, suggesting that chlorogenic acid could be responsible for the increase in the Treg population and the suppressive activity in our study [41]. Additionally, theobromine, the second most common molecule found in our YM extract (892 ± 55 mg/Kg), has been identified as the main cause of the immunomodulatory effects of cocoa in rats [42]. Considering that theobromine inhibits poly [ADP-ribose] polymerase 1, a nuclear enzyme involved in DNA repair that negatively regulates Treg function [43,44], it is possible to argue that theobromine could also contribute to the increased suppressive Treg activity by YM observed in vitro. However, more studies are necessary to specifically evaluate the contributions of the different compounds present in the YM over the observed protection against EAE and the effect over Tregs. Additionally, since other immune cells are important mediators in MS, it is possible that YM could target different immune cell types in addition to Tregs. In fact, we have recently found that YM modulates macrophage polarization, increasing this anti-inflammatory macrophage population, which could also mediate certain protective effects noted in our EAE model by YM [17].

Immune cell infiltration into the brain and spinal cord is one of the key steps in neuroinflammatory conditions, such as MS. In fact, the blood–brain barrier (BBB) plays an important role in regulating the flow of immune cells between the blood and the brain, and its integrity is compromised during MS [45]. BBB integrity could be affected by different factors, including reactive oxygen species (ROS) production, as ROS are highly produced during MS [46]. Interestingly, chlorogenic acid can cross the BBB and inhibit ROS production, improving BBB integrity and reducing peripheral immune cell infiltration, which could explain the reduced peripheral B and T cell infiltration into the brains of animals treated with YM [47,48]. Additionally, matrix metalloproteinases (MMPs), which are almost undetectable in normal adult brain but highly expressed during neuroinflammatory processes, such as MS, could also compromise BBB integrity by attacking the basal lamina macromolecules that line the blood vessels [49,50,51]. Accordingly, it has been recently shown that chlorogenic acid is capable of reducing MMP expression, improving BBB integrity in a murine model of intracerebral hemorrhage [52]. Considering the high levels of chlorogenic acid in our YM infusion, we can speculate that the reduced peripheral immune cell infiltration into the brain parenchyma observed in the animals treated with YM could be explained in part by the improved BBB integrity induced by chlorogenic acid that decrease ROS production and MMP expression in the CNS, although further experiments evaluating BBB integrity and MMPs expression are necessary to test this idea.

Even though MS prevalence is growing worldwide according to the Atlas of MS, with a rise of 30% in recent years, different regions have differently growing rates. For example, in the US, the MS prevalence is 309.2 cases per 100,000 inhabitants, with approximately 400,000 people suffering from MS [53]. In Europe, the prevalence is 142.81 cases per 100,000 inhabitants, with more than 700,000 people suffering this disease [54]. In contrast, in South American countries, the MS prevalence is significantly lower, ranged among 0.75 to 38.2 cases per 100,000 inhabitants [55]. For example, the prevalence rate in Chile has been found to be 5.69 per 100,000 people [56], while in Argentina the prevalence is around 18 per 100,000 inhabitants [57]. Although multiple factors, such as genetics, social determinants, and environmental factors, could play essential roles in the geographic distribution of MS, differences in diets, a fundamental lifestyle factor, could contribute to these differences. In fact, findings from a recent study suggest that the type of diet adopted is a key risk factor associated with the relapse of MS [58]. Interestingly, countries in South America with the lowest prevalence are the predominant consumers of YM, with Uruguay distinguishing itself with an annual per capita consumption that surpasses 8 kg, followed by Argentina, where the average consumption per person reaches 6.5 kg annually [59]. Although many other differences arise between the different regions, it is possible that the consumption of YM in South America contributes to the reduced prevalence of MS in this region. Further studies will be necessary to evaluate whether YM consumption reduces symptoms in MS patients.

Some strengths and limitations of our work should be considered. The consistency of our results regarding the reduction in clinical symptoms, decreased incidence, and diminished immune cell infiltration, together with the different techniques used to corroborate the reduced immune cell infiltration and our in vitro approach, reinforce the idea of the protective effect of YM over the EAE symptoms. Our study has also some limitations. First, while we noted that the preventive use of YM led to a decrease in EAE, the therapeutic application of YM was not assessed. Second, although we observed increased levels of Tregs after YM administration in vivo, we did not detect any influence of YM on Treg differentiation in vitro, suggesting that YM could be modulating also other cell types that affect Treg expansion. Third, despite the potent effect of YM on EAE symptoms, YM compounds were not analyzed separately; therefore, we do not know which compound(s) was/were responsible for this effect. Fourth, even though we observed an important reduction in EAE symptoms and incidence in animals receiving YM, we did not perform a parallel experiment using any regular drug with known immunosuppressive capability, in order to compare the effect of YM versus this standard treatment. Thus, future studies specifically evaluating the different compounds of YM and their effect in EAE, the therapeutical capacity of YM versus a regular treatment, and the analysis of other immune cell types susceptible to being modulated by YM are necessary to have a more complete picture of the effect of YM over the immune system and its therapeutic impact in MS.

## 5. Conclusions

**Figure 6:**
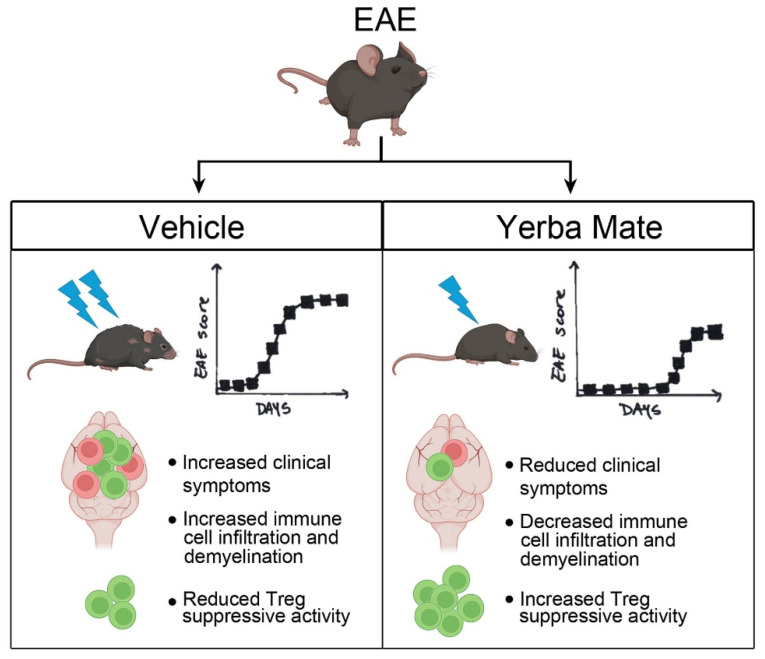
Summary diagram of the main findings. In the EAE mouse model, YM treatment reduces clinical symptoms, histological hallmarks, immune cell infiltration, and demyelination, as well as increasing the Treg suppressive capacity in vitro.

In summary, we have shown that YM consumption effectively reduced the symptoms and incidence observed in a murine model of MS. Reduced peripheral immune cell infiltration into the CNS and myelin damage were observed in the animals with prophylactic administration of YM. Accordingly, YM consumption increased the Treg population in peripheral organs and also improved their suppressive activity in vitro. All these results suggest that this medicinal herb, in part by targeting Treg population, reduces EAE symptoms and could be considered as a complementary treatment for MS (Figure 6). Considering that the consumption of YM in some South American countries is estimated at around 12–23 g/day with a per capita consumption of YM around 8–10 kg/year in Uruguay [60] and that our study used a high dose of YM but over a short period of time, it is possible to speculate that the continuous consumption of YM could have beneficial effects in MS patients. Accordingly, we have started a clinical trial to evaluate the effect of YM consumption in Chilean MS patients.

## Data Availability

All data are presented in the manuscript and are available upon request due to due to privacy reasons.

## Supplementary Materials

The following supporting information can be downloaded at: https://www.mdpi.com/article/10.3390/nu17050897/s1, Video S1: EAE-treated mice receiving yerba mate appear healthier than the EAE group receiving vehicle.

## Abbreviations

The following abbreviations are used in this manuscript:

YM	Yerba mate
MS	Multiple sclerosis
EAE	Experimental autoimmune encephalomyelitis
Tregs	Regulatory T cells
CNS	Central nervous system
FMT	Fecal microbiota transplantation
DSS	Dextran sodium sulfate
CTV	Cell Trace Violet
SLOs	Secondary lymphoid organs
BBB	Blood–brain barrier
ROS	Reactive oxygen species
MMPs	Matrix metalloproteinases
